# Supplementary material for: Effect of Iron Complex Source on MWWTP Effluent Treatment by Solar Photo-Fenton: Micropollutant Degradation, Toxicity Removal and Operating Costs
Source: Molecules. 2022 Aug 27;27(17):5521. doi: 10.3390/molecules27175521 (PMC9458207; doi:10.3390/molecules27175521)
Supplement: Supplementary file 1 [file molecules-27-05521-s001.zip › molecules-1879361-supplementary.pdf]

Supplementary Material

Effect of Iron Complex Source on MWWTP Effluent Treatment by Solar Photo-Fenton: Micropollutant Degradation, Toxicity Removal and Operating Costs

Eduardo O. Marson [1], Ivo A. Ricardo [1,2], Cleiseano E. S. Paniagua [1], Serena M. Malta [3], Carlos Ueira-Vieira [3], Maria Clara V. M. Starling [4], José Antonio Sánchez Pérez [5] and Alam G. Trovó [1,5,*]

[1] Instituto de Química, Universidade Federal de Uberlândia, Uberlândia 38400-902, Brazil
[2] Faculdade de Ciências Naturais e Exactas, Universidade Save, Chongoene 0301-01, Gaza, Mozambique
[3] Instituto de Biotecnologia, Universidade Federal de Uberlândia, Uberlândia 38405-319, MG, Brazil
[4] Departamento de Engenharia Sanitária e Ambiental, Universidade Federal de Minas Gerais, Belo Horizonte 31270-010, Brazil
[5] Solar Energy Research Centre (CIESOL), University of Almería, Ctra. de Sacramento s/n, ES04120 Almería, Spain
* Correspondence: alamtrovo@ufu.br; Tel.: +55-34-3291-6380

Table S1. Physicochemical characterization of municipal wastewater treatment plant effluent sample.

Parameter	Unit	Value
pH	-	6.9
Color	mg L^{-1} Pt/Co	440
Turbidity	NTU	47.5
Total solids	mg L^{-1}	601
Dissolved solids	mg L^{-1}	523
Suspended solids	mg L^{-1}	36
Conductivity	µS cm^{-1}	1040
Chemical oxygen demand	mg O_2 L^{-1}	200
Biochemical oxygen demand	mg O_2 L^{-1}	80
Alkalinity	mg $CaCO_3$ L^{-1}	312
Chloride	mg L^{-1}	200
Iron	mg L^{-1}	0.7
Total carbon	mg C L^{-1}	95.3
Inorganic carbon	mg C L^{-1}	57.2
Organic carbon	mg C L^{-1}	38.1

Table S2. Reagents prices (iron, chelating agents and oxidant) for the photo-Fenton process, as according to Sigma-Aldrich®.

Reagent	Price (US$ kg^{-1})
$Fe(NO_3)_3.9H_2O$	137
Cit	86
EDDS	2984
EDTA	206
NTA	173
H_2O_2	14

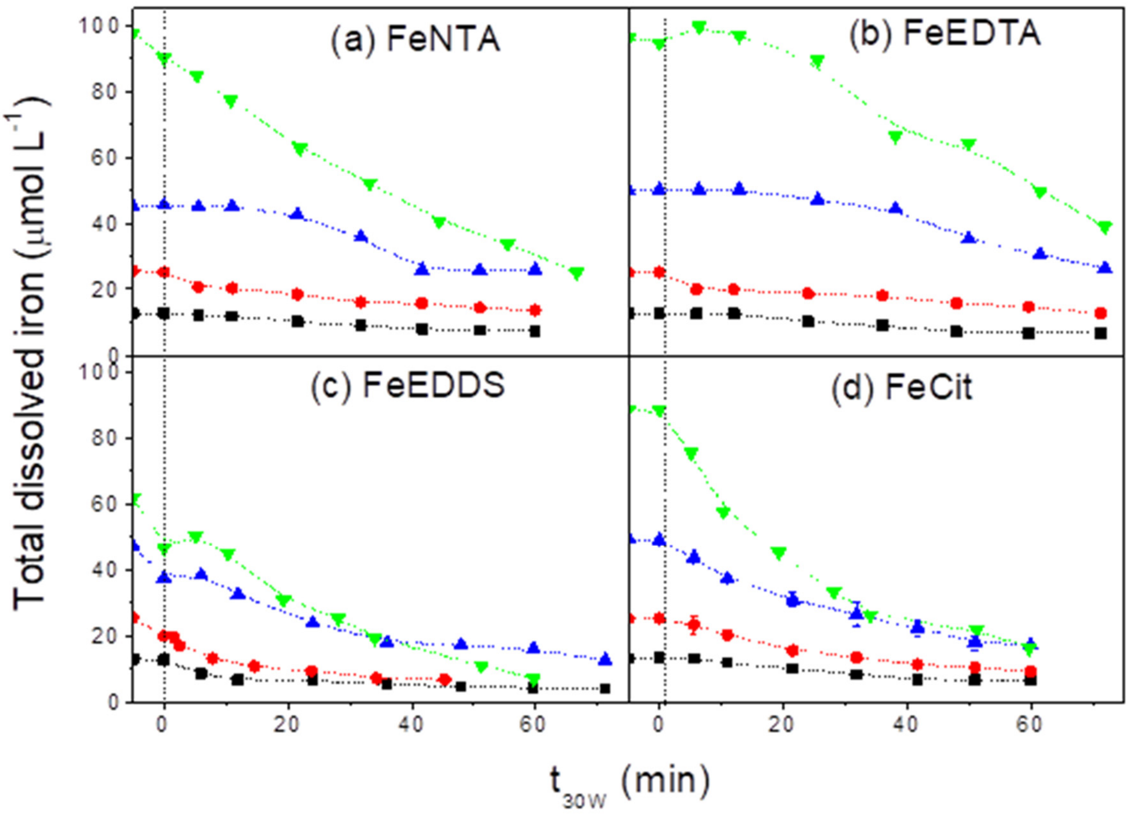

Figure S1. Influence of iron complex source and concentrations (■ 12.5 μmol L⁻¹, ● 25 μmol L⁻¹, ▲ 50 μmol L⁻¹ and ▼ 100 μmol L⁻¹) on total dissolved iron concentration during the degradation of benzophenone-3, fipronil and propylparaben by solar photo-Fenton process using (a) FeNTA, (b) FeEDTA, (c) FeEDDS and (d) FeCit. Initial conditions: [CEC]= 100 μg L^{-1} (for each compound), [H_2O_2] = 5.9 mmol L^{-1}, Fe:L= 1:1; pH= 6.9 (natural of the MWWTP effluent).

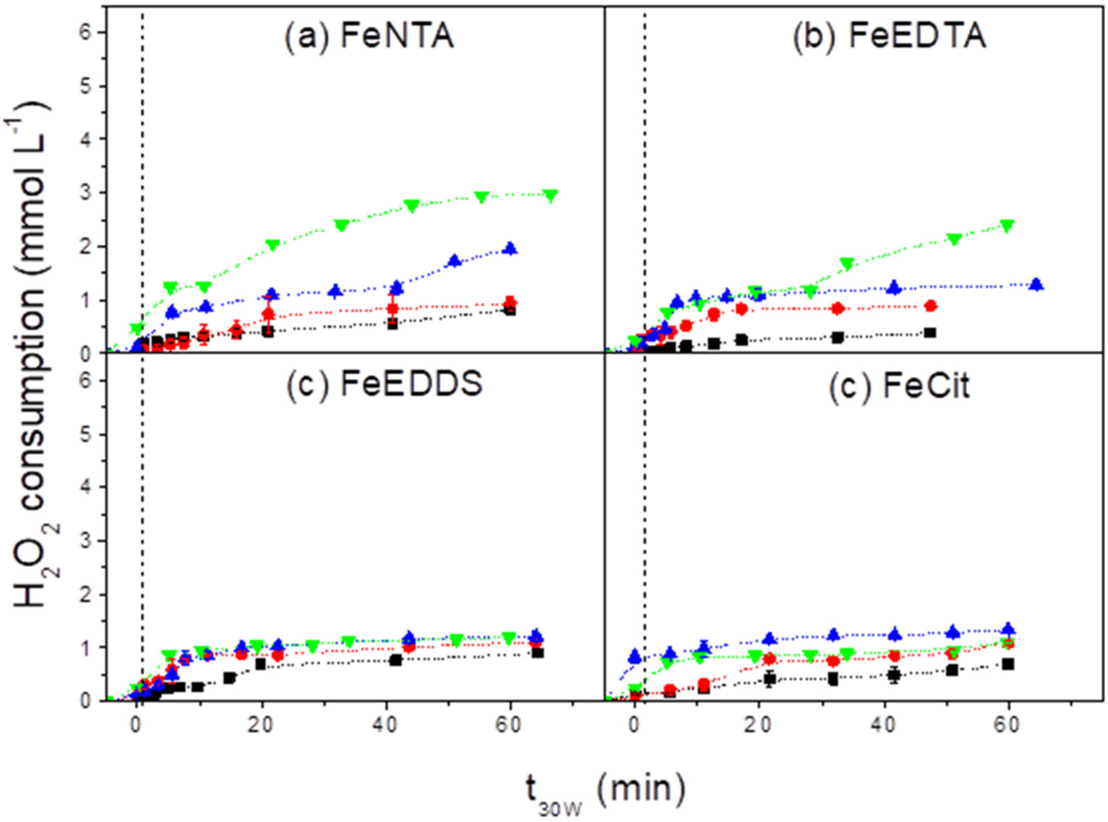

Figure S2. Influence of iron complex source and concentrations (■ 12.5 µmol L⁻¹, ● 25 µmol L⁻¹, ▲ 50 µmol L⁻¹ and ▼ 100 µmol L⁻¹) on H_2O_2 consumption during the degradation benzophenone-3, fipronil and propylparaben by solar photo-Fenton process using the iron complexes (a) FeNTA, (b) FeEDTA, (c) FeEDDS and (d) FeCit. Initial conditions: [CEC] = 100 µg L⁻¹ (for each compound), [H_2O_2] = 5.9 mmol L⁻¹, Fe:L= 1:1; pH= 6.9 (natural of the MWWTP effluent).

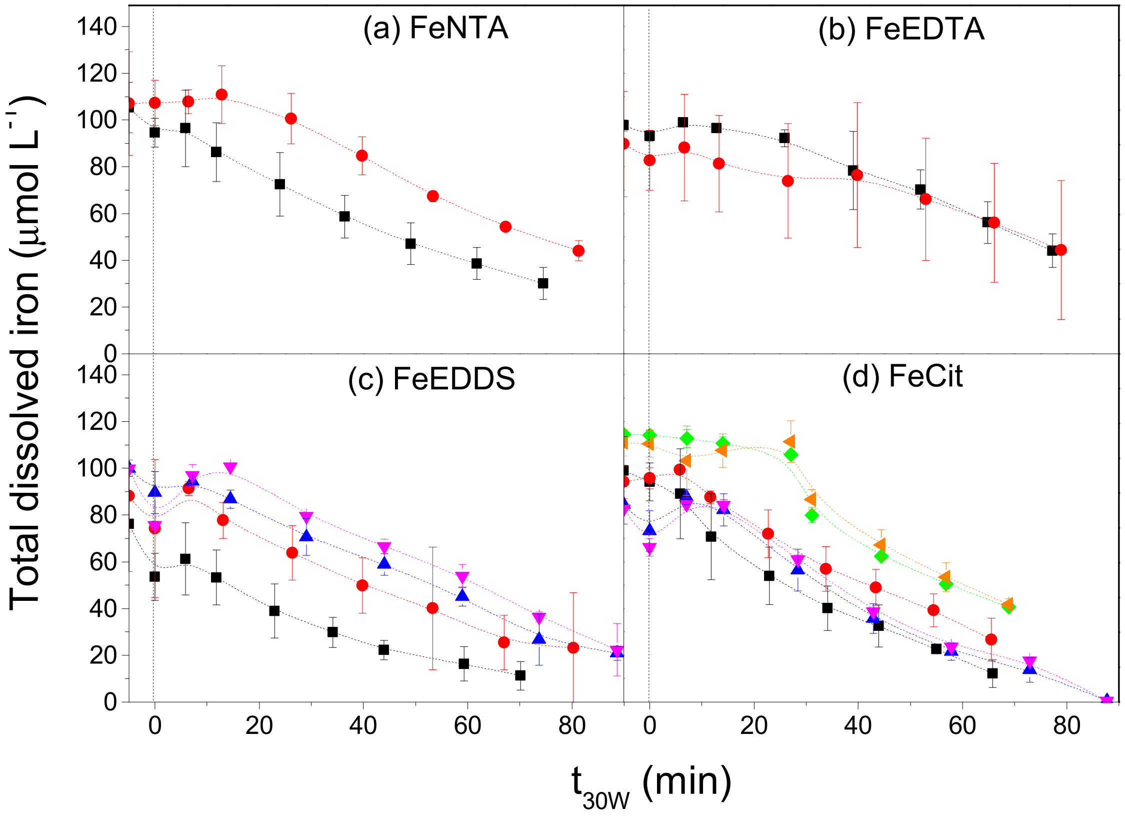

Figure S3. Influence of Fe/L molar ratio (■ 1:1, ● 1:2, ▲ 1:3, ▼ 1:4, ♦ 1:5 and ◀ 1:6) on total dissolved iron concentration during the degradation of benzophenone-3, fipronil and propylparaben by solar photo-Fenton process using the iron complexes (a) FeNTA, (b) FeEDTA, (c) FeEDDS and (d) FeCit. Initial conditions: [CEC]= 100 μg L^{-1} (for each compound), [H_2O_2] = 5.9 mmol L^{-1}, [Fe^{3+}] = 100 μmol L^{-1}; pH= 6.9 (natural of the MWWTP effluent).

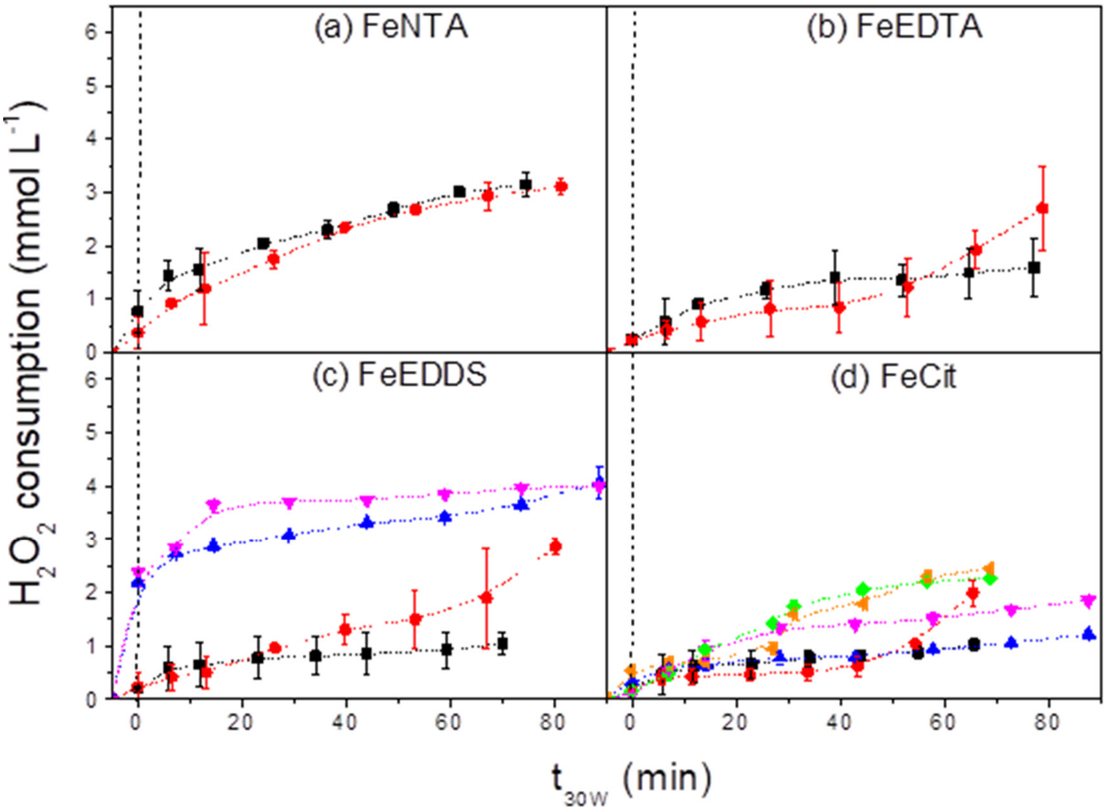

Figure S4. Influence of Fe/L molar ratio (■ 1:1, ● 1:2, ▲ 1:3, ▼ 1:4, ♦ 1:5 and ◀ 1:6) on H_2O_2 consumption during the degradation of benzophenone-3, fipronil and propylparaben by solar photo-Fenton process using the iron complexes (a) FeNTA, (b) FeEDTA, (c) FeEDDS and (d) FeCit. Initial conditions: [CEC]= 100 μg L^{-1} (for each compound), [H_2O_2] = 5.9 mmol L^{-1}, [Fe^{3+}] = 100 μmol L^{-1}; pH= 6.9 (natural of the MWWTP effluent).

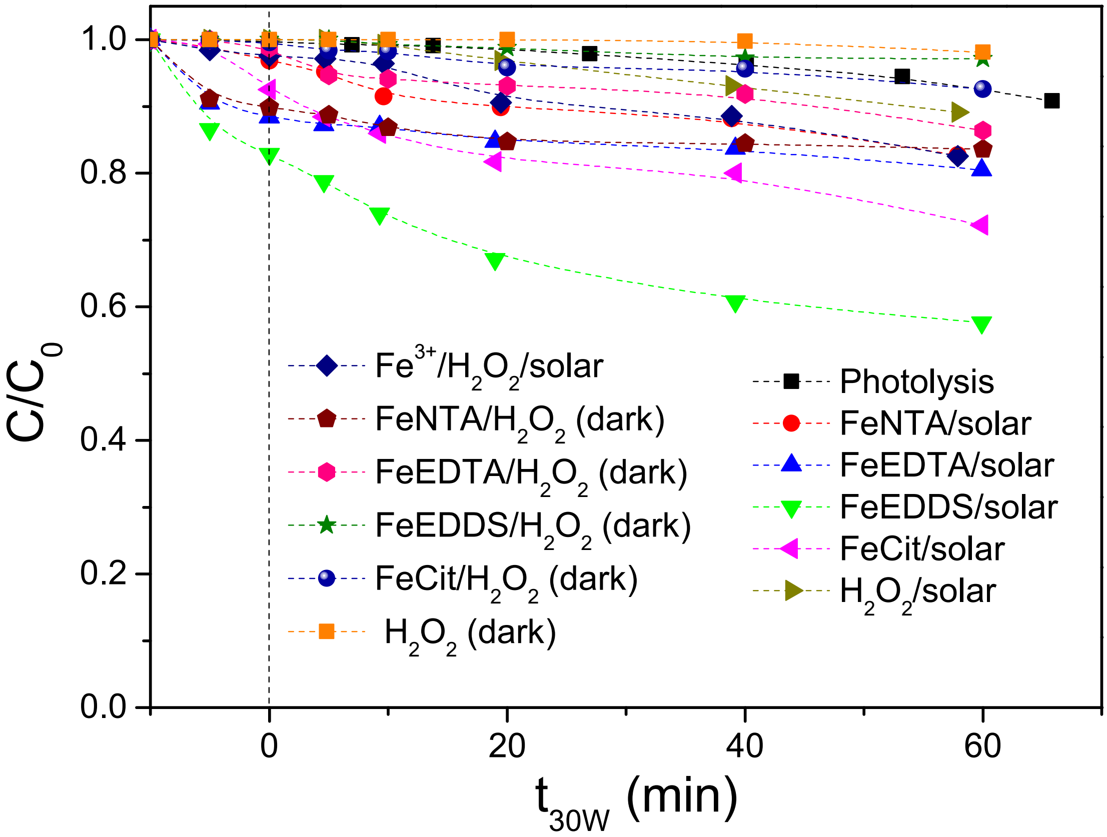

Figure S5. Control experiments for the degradation benzophenone-3, fipronil and propylparaben using the iron complexes FeNTA, FeEDTA, FeEDDS and FeCit. Initial conditions: [CEC]= 100 µg L^{-1} (for each compound), Fe:L= 1:1 (FeEDTA and FeNTA); 1:3 (FeEDDS) and 1:5 (FeCit), [Fe^{3+}]= 100 µmol L^{-1}; [H_2O_2]= 1.47 mmol L^{-1} (for FeEDDS), 2.94 mmol L^{-1} (for FeCit, Fe^{3+}, H_2O_2 (in the dark) and H_2O_2/solar) and 5.9 mmol L^{-1} (for FeEDTA and FeNTA), pH= 6.9 (natural of the MWWTP effluent).

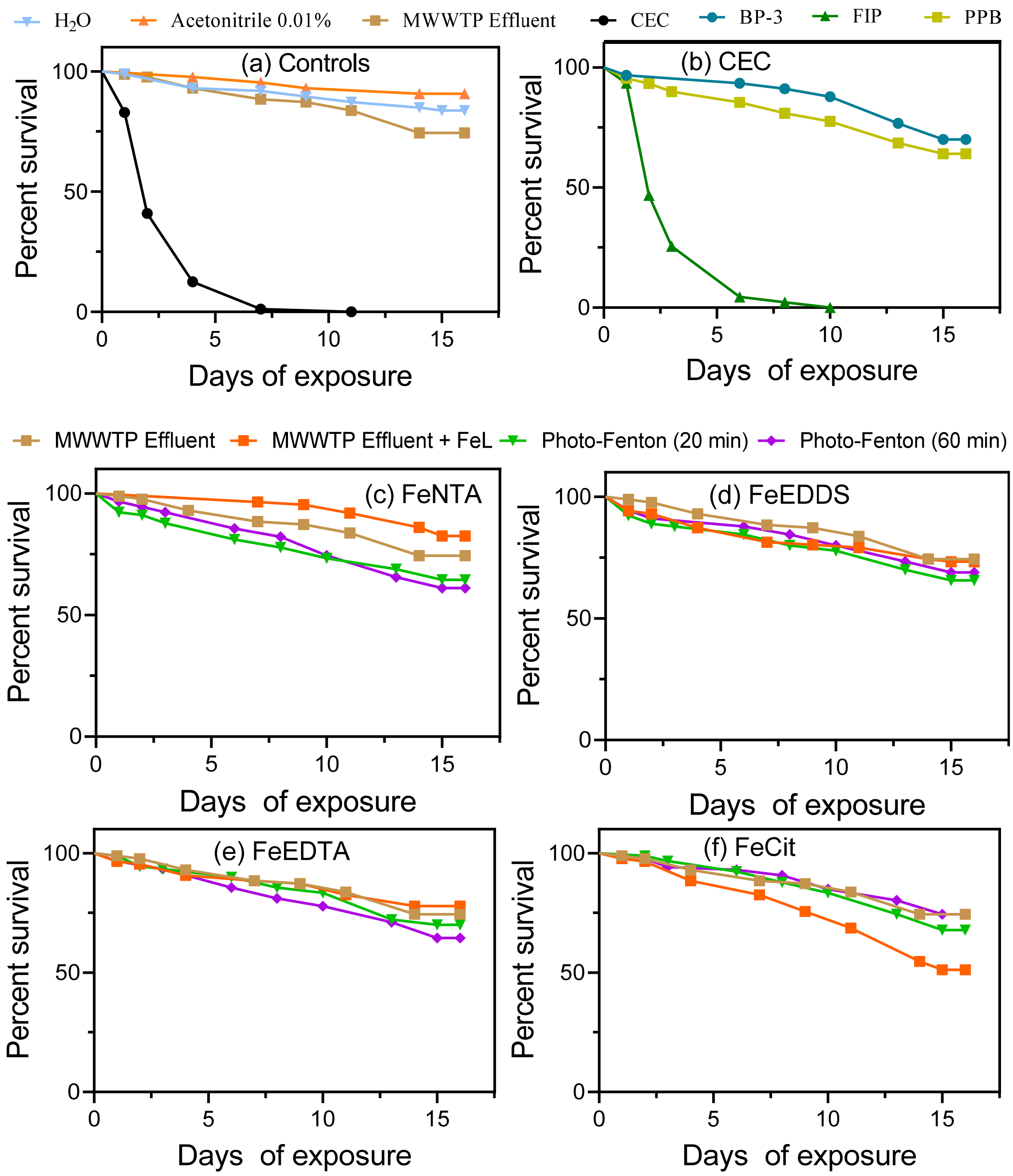

Figure S6. Results obtained during *D. melanogaster* survival assay for (a) Control experiments; (b) individual solutions of the target-compounds and (c, d, e and f) samples of MWWTP in the absence of benzophenone-3, fipronil and propylparaben but in the presence of iron complexes before and after solar/photo-Fenton treatment. Initial conditions: [CEC]= 100 µg L^{-1} (for each compound), Fe/L= 1:1 (FeEDTA and FeNTA); 1:3 (FeEDDS) and 1:5 (FeCit), [Fe^{3+}]= 100 µmol L^{-1}; [H_2O_2]= 1.5 mmol L^{-1} (for FeEDDS), 2.94 mmol L^{-1} (for FeCit, Fe^{3+}, H_2O_2 (in the dark) and H_2O_2/solar) and 5.9 mmol L^{-1} (for FeEDTA and FeNTA), pH= 6.9 (natural of the MWWTP effluent).

Bioassays with D. Melanogaster

Acute toxicity assays were performed for non-treated and treated solutions using *Drosophila melanogaster (D. melanogaster)*. All bioassays were performed in triplicates.

D. melanogaster, an invertebrate also termed as fruit fly, has some desirable characteristics (short lifespan, fully known genome sequence and ease of genetic manipulation) that are not characteristic of higher organisms and stimulate its use as a model for acute toxicity assays. Furthermore, metabolic pathway for the metabolism of environmental toxins observed in this organism is similar to that presented by vertebrates, including humans. Lifespan of *D. melanogaster* was used to assess acute toxicity in many studies, using leachates from municipal solid wastes, MWWTP effluent containing pesticides, aqueous solutions of parabens and antibiotics [1,2].

In this study, Canton S. strain flies were used for the acute toxicity test, in which mortality between 0-4 days old wild-type *D. melanogaster* was monitored. The mortality of flies was evaluated for initial solution of MWWTP effluent containing BP-3, FIP and PPB (100 μg L^{-1} each), and for photo-Fenton (20 and 60 min) processes evaluated under better experimental conditions for FeCit, FeEDDS, FeEDTA and FeNTA. For each bioassay, 30 flies (n = 30) (15 females and equal number of males) were exposed to of treated and untreated samples by mixing samples to fly food. The food was made in a medium enriched with mashed potato (1.5 g, 75% instant mashed potato, 15% yeast extract, 9.3% glucose, 0.07% nipagin) and 5 mL of each sample of the treated and untreated solutions. Fly food was replaced every 2 to 3 days and the number of dead flies was registered until the 15 days corresponding to the experiment.

For control experiments, fly lifespan was assessed when exposed to distilled water, acetonitrile (0.01%), MWWTP effluent (in the absence of target-compounds) and MWWTP effluent degradation samples (at the same sampling time for target-compounds degradation, 20 and 60 min) using the FeCit, FeEDDS, FeEDTA and FeNTA systems were also verified. In addition, solutions containing only FIP or PPB or BP-3 in the MWWTP effluent were also tested. Flasks containing flies were kept at 25 ºC in a light/dark cycle (12 h/12 h) during experiments. Mean life span was calculated using the Kaplan-Meier test using the *Graph Pad Prism* 8.02 software.

References

1. Gomes Júnior, O.; Batista, L.L.; Ueira-Vieira, C.; Sousa, R.M.F.; Starling, M.C.V.M.; Trovó, A.G. Degradation Mechanism of Fipronil and Its Transformation Products, Matrix Effects and Toxicity during the Solar/Photo-Fenton Process Using Ferric Citrate Complex. *J. Environ. Manage.* **2020**, *269*, 110756, doi:10.1016/j.jenvman.2020.110756.
2. Gonçalves, B.R.; Guimarães, R.O.; Batista, L.L.; Ueira-Vieira, C.; Starling, M.C.V.M.; Trovó, A.G. Reducing Toxicity and Antimicrobial Activity of a Pesticide Mixture via Photo-Fenton in Different Aqueous Matrices Using Iron Complexes. *Sci. Total Environ.* **2020**, *740*, 140152, doi:10.1016/j.scitotenv.2020.140152.
